# The Effects of Cannabidiol on Aqueous Humor Outflow and Trabecular Meshwork Cell Signaling

**DOI:** 10.3390/cells11193006

**Published:** 2022-09-27

**Authors:** Alyssa S. Aebersold, Zhao-Hui Song

**Affiliations:** Department of Pharmacology and Toxicology, School of Medicine, University of Louisville, Louisville, KY 40292, USA

**Keywords:** cannabidiol, aqueous humor outflow, trabecular meshwork

## Abstract

Intraocular pressure (IOP) is regulated primarily through aqueous humor production by ciliary body and drainage through uveoscleral and trabecular meshwork (TM) tissues. The goal of this study was to measure the effect of non-psychotropic cannabidiol (CBD) on aqueous humor outflow through TM and assess the effect of CBD on the TM cell signaling pathways that are important for regulating outflow. Perfused porcine eye anterior segment explants were used to investigate the effects of CBD on aqueous humor outflow. Cultured porcine TM cells were used to study the effects of CBD on TM cell contractility, myosin light chain (MLC) and myosin phosphatase targeting subunit 1 (MYPT1) phosphorylation, and RhoA activation. In the anterior segment perfusion experiments, aqueous humor outflow was increased significantly within 1 h after adding 1 µM CBD and the effect was sustained over the 5 h of measurement. Treatment of TM cells with 1 µM CBD significantly decreased TM cell-mediated collagen contraction, inhibited phosphorylation of MLC and MYPT1, and reduced RhoA activation. Our data demonstrate, for the first time, that as a potential therapeutic agent for lowering intraocular pressure, CBD can enhance aqueous humor outflow and modify TM cell signaling.

## 1. Introduction

Glaucoma is one of the leading causes of human blindness, with chronically elevated intraocular pressure (IOP) as the major risk factor [1,2,3]. IOP is regulated through a dynamic balance of aqueous humor secretion by the ciliary body and outflow via the trabecular meshwork (TM) and uveoscleral routes [1,2,3]. Therefore, IOP can be lowered by decreasing aqueous humor formation or increasing aqueous outflow (through either TM or uveoscleral routes). Current pharmacotherapies for glaucoma include prostaglandin analogs, β-adrenoceptor antagonists, α-adrenoceptor agonists, carbonic anhydrase inhibitors, cholinergic agonists, and most recently, Rho kinase inhibitors [4,5,6,7,8]. However, existing drugs have side-effects and patients frequently become tolerant to the drugs over the course of their disease (available drugs no longer lower IOP). Therefore, there is an urgent need for novel drugs to lower IOP through novel mechanisms of action with fewer side effects [4,5,6,7,8].

Cannabidiol (CBD) is one of the major active constituents of cannabis [9,10]. Unlike Δ^9^-tetrahydrocannabinol (Δ^9^-THC), CBD is non-psychotropic [9,10]. CBD has a wide range of therapeutic potentials, including the treatment of cancer [11], inflammatory diseases [12], neurodegenerative diseases [13,14], and psychiatric diseases [15]. Recently, CBD (Epidiolex/Epidyolex)) received both food and drug administration (FDA) and European Medicines Agency (EMA) approvals for treating epilepsy in children; this is the first time a constituent isolated from cannabis has been FDA approved [16].

The observation that smoked marijuana lowers IOP was first noted more than 50 years ago [17]. Since then, many papers have demonstrated that various phytocannabinoids, including Δ^9^-THC and CBD, are able to lower IOP, suggesting their potentials as therapeutic agents for glaucoma [18,19,20]. However, Δ^9^-THC has psychotropic side-effects which limit its clinical use for glaucoma [18,20]. Since CBD is non-psychotropic, its IOP-lowering effects have more therapeutic potential.

The Rho/Rho kinase signaling pathway is a major regulator of conventional aqueous humor outflow through the TM [21,22,23,24,25]. This pathway regulates phosphorylation of downstream regulators regulatory myosin light chain (MLC) and myosin phosphatase target subunit 1 (MYPT1) [26]. By preventing the phosphorylation of MLC and MYPT1, Rho kinase inhibitors such as Rhopressa (Netarsudil) and Galantec (Ripasudil), inhibit TM tissue contraction and enhance aqueous humor outflow through the TM [21,22,23,24,25,27,28,29].

Despite evidence that CBD reduces IOP, the underlying molecular mechanism has not been elucidated. One potential mechanism is that CBD enhances aqueous outflow in the TM, but this has not been studied. In this study we first investigated if CBD modulates aqueous humor outflow using perfused porcine anterior segments. We then investigated whether CBD alters the contractility of cultured porcine TM cells and the involvement of the Rho/Rho kinase signaling pathways in the effects of CBD on TM cells. We chose 1 μM concentration of CBD for this study because it has been shown that at this concentration CBD acts specifically on its multiple molecular targets to produce its pharmacological effects [30].

## 2. Materials and Methods

### 2.1. Porcine Anterior Segment–Perfused Organ Culture Model

It has been shown that in the anterior segment perfused organ culture model, flow rates are physiological (approximately 2.75 µL/min) and outflow is through the TM [31,32,33,34,35]. Porcine anterior segment perfused organ culture was performed as previously described [31,32,33,34,35]. Within 30 min of decapitation, fresh porcine eyes were collected from a local slaughterhouse. Ciliary body and iris were carefully removed, and anterior segment explants were prepared to contain the undisturbed TM, a 2 to 5 mm rim of sclera and intact cornea. Subsequently, the porcine anterior segments were mounted in a custom-made perfusion culture apparatus and perfused with Dulbecco’s modified Eagles medium (DMEM). Organ cultures were maintained with 5% CO_2_ at 37 °C. A constant perfusion head of 10 cm (~7.35 mm Hg) was used for perfusion and outflow was stabilized for at least 5 h. For CBD or vehicle experiments, the explants that stabilized between 1.5 and 8 µL/min at 7.35 mmHg were used. After stabilization, CBD (Cayman Chemical, Ann Arbor, MI, USA) was introduced. The anterior segments were then perfused continuously with CBD-containing medium for 5 h, and the outflow was monitored. Medium containing no CBD was run in parallel. Aqueous humor outflow was calculated as the rate of flow of perfusate (in microliters per minute). Drug effects were evaluated in each explant as the percentage change of outflow in drug-treated eyes over pre-drug baseline outflow. Ten eyes were used for each group of treatment.

### 2.2. Porcine Trabecular Meshwork Cell Cultures

Previously published methods were adapted to culture primary porcine TM cells [36,37]. Briefly, blunt dissections were used to isolate TM from fresh porcine eyes. TM cells were cultured in DMEM with 5% CO_2_ at 37 °C. In addition to their morphology, the identity of TM cells was established by their ability to secrete tissue plasminogen activator and to take up acetylated low-density lipoprotein and [36,37].

### 2.3. Collagen Gel Contraction Assays

Collagen gel contractility assays were performed following previously published procedures [38]. The wells of 24-well cell culture plates (Corning, Corning, NY, USA) were each coated with 1 mL 1% bovine serum albumin (BSA) for 1 h at 37 °C. Porcine TM cells were collected by treatment of cultures with trypsin-ethylenediaminetetraacetic acid (EDTA), washed with 1× PBS, and re-suspended in DMEM at a density of 1 × 10^6^ cells/mL. Rat tail collagen type I (Santa Cruz Bio, Dallas, TX, USA), 10X phosphate buffered saline (PBS), sterile dH_2_O, and 1 N NaOH were mixed at ratios following manufacturer instructions to obtain a final concentration of 1.9 mg/mL collagen and final cell density of 2 × 10^5^ cells/mL. The resultant mixture was added to BSA coated wells (1% BSA, 0.5 mL/well). Collagen gels were allowed to polymerize at 37 °C with 5% CO_2_ for 90 min. Once released from the wells, serum-free DMEM (0.5 mL), with or without CBD, was added to the gels. The gels were imaged at 48 h. The area of the collagen gels was calculated using ImageJ software (National Institutes of Health, Bethesda, MD, USA). For normalization, the area of the collagen gel containing vehicle treated TM cells was set at 100%, and the changes in the area for CBD treatment are shown as a bar graph representing the mean ± SEM (*n* = 5).

### 2.4. Western Blot Analysis

Porcine TM cell proteins were prepared as previously described [39]. After incubating at 100 °C with 2× Laemmli buffer under reducing conditions for 20 min, protein bands were resolved on a 10% sodium dodecyl sulfate (SDS)-polyacrylamide gel. Subsequently, protein bands were transferred onto a nitrocellulose membrane and the membranes was incubated for 1 h in 5% nonfat milk in Tris-buffered saline with Tween^®^ 20 (TBS-T) (10 mM Tris-HCl, 150 mM NaCl, and 0.3% Tween 20, pH 8.0). The membrane was thenincubated overnight at 4 °C with primary anti-glyceraldehyde-3-phosphate dehydrogenase (anti-GAPDH) and anti-phospho-MLC2 (Ser19) or anti-phospho-MYPT1 (Thr853) antibody (Cell Signaling, Beverly, MA, USA). Subsequently, the membranes were washed three times with TBS-T buffer for 5 min, and then incubated with horseradish peroxidase (HRP)–conjugated secondary antibody (Santa Cruz Biotechnology, Dallas, TX, USA) at room temperature for 1 h. The membrane was then washed with TBS-T buffer for three times (each time 5 min) and the antibody-bound protein bands were developed using enhanced chemiluminescence Western blotting substrate from Pierce™ (Fisher Scientific, Waltham, MA, USA).

### 2.5. RhoA Activation Assay

Samples were collected and assays were performed according to manufacturer’s (Cytoskeleton Inc., Denver, CO, USA) instructions. Cells were grown to confluence and serum starved overnight. Cells were then treated with vehicle or CBD, lysed on ice using lysis buffer (50 mM Tris, 10 mM MgCl_2_, 0.5 M NaCl, 0.1% Triton X-100, and 0.1% SDS, pH 7.5) containing a protease inhibitor cocktail (Sigma, St. Louis, MO, USA), then collected into pre-chilled 1.5 mL microcentrifuge tubes. Cell lysates were then centrifuged at 10,000× *g* at 4 °C, and supernatant containing 50 µg of protein was incubated with 10 µg Rhotekin-RBD beads (Cytoskeleton, Denver, CO, USA) in a final volume of 300 µL for 1 h at 4 °C. Following incubation, beads were washed with ice-cold wash buffer and centrifuged at 5000× *g*. The immunoprecipitated complex was re-suspended in 2× SDS sample buffer, boiled at 100 °C for 5 min, and then subjected to 10% SDS- polyacrylamide gel electrophoresis, followed by Western blot analysis. The separated proteins were immunoblotted with antibody against RhoA (Cytoskeleton Inc., Denver, CO, USA).

### 2.6. Data Analysis

For anterior segment perfusion studies, results are presented as changes in aqueous humor outflow (% of basal) mean ± SEM. The level of significance was chosen as *p* < 0.05. For the collagen gel contraction assay, images of gel areas were quantified with the use of ImageJ program (NIH, Bethesda, MD, USA). For Western blot assays, the bands on X-ray films were scanned by the Epson Perfection V39 (Epson, Long Beach, CA, USA) and were quantified with the use of ImageJ. All data are analyzed and plotted with Prism software (GraphPad, San Diego, CA, USA). Unpaired two-tailed Student’s *t*-tests were used to compare the mean ± SEM of CBD and vehicle treatment groups. The level of significance for all studies was set at *p* < 0.05.

## 3. Results

### 3.1. The Effects of CBD on Aqueous Humor Outflow

**Figure 1:**
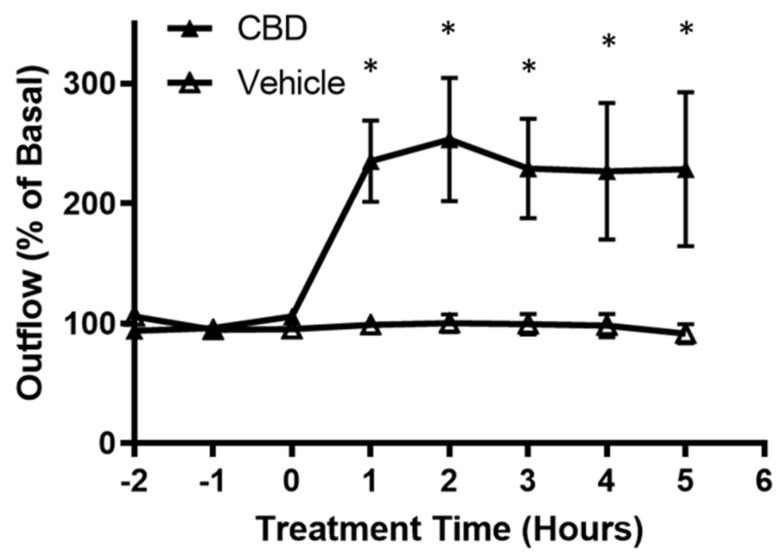
Effects of cannabidiol on aqueous humor outflow. Application of 1 µM CBD caused a significant increase in aqueous humor outflow lasting from 1 h to 5 h. Basal outflow is defined as the average outflow of the three time points prior to CBD administration. Results are expressed as mean ± SEM; *n* = 10. * Significant differences between 1 µM CBD and vehicle (DMEM + 0.001% ethanol) determined by *t*-test, *p* < 0.05.

Aqueous humor outflow studies were performed using the porcine anterior segment perfused organ culture model. As shown in Figure 1, the application of 1 µM CBD more than doubled aqueous humor outflow at 1 h after treatment when compared with vehicle. This effect lasted for the measurement window of 5 h.

### 3.2. The Effect of CBD on Collagen Gel Contraction Mediated by TM Cells

**Figure 2:**
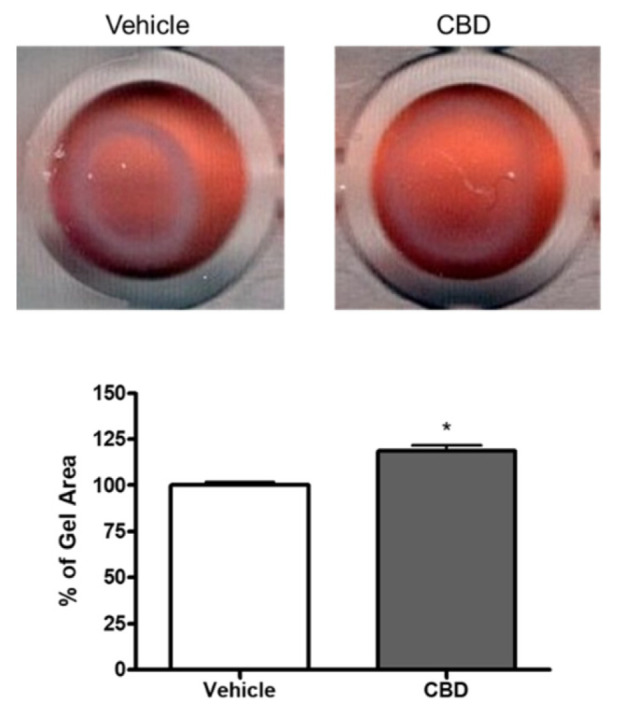
Effect of cannabidiol on collagen gel contraction mediated by trabecular meshwork cells. Cells grown embedded in collagen gels were treated with DMEM or DMEM containing 1 µM CBD for 48 h. Top: Representative photographs of collagen gel cultures of TM cells incubated for 48 h with the indicated drug treatment. Bottom: The mean ± SEM of results of five experiments are shown. Treatment with CBD significantly opposed the basal level of gel contraction. * Significant difference from vehicle determined by *t*-test, *p* < 0.05.

To determine if collagen contraction mediated by TM cells is altered by CBD, TM cells were cultured in a three-dimensional collagen gel, and change in area of the gel in response to DMEM (vehicle) or DMEM containing CBD (1 µM) treatment was measured. As shown in Figure 2, vehicle treated TM cells caused collagen contraction. Importantly, CBD treatment significantly opposed TM cell-mediated gel contraction (CBD gel area 118.7% ± 3.042 compared to vehicle, *n* = 5; *p* < 0.05). These results demonstrate that CBD relaxes TM cell contraction.

### 3.3. The Effects of CBD on MLC Phosphorylation in TM Cells

**Figure 3:**
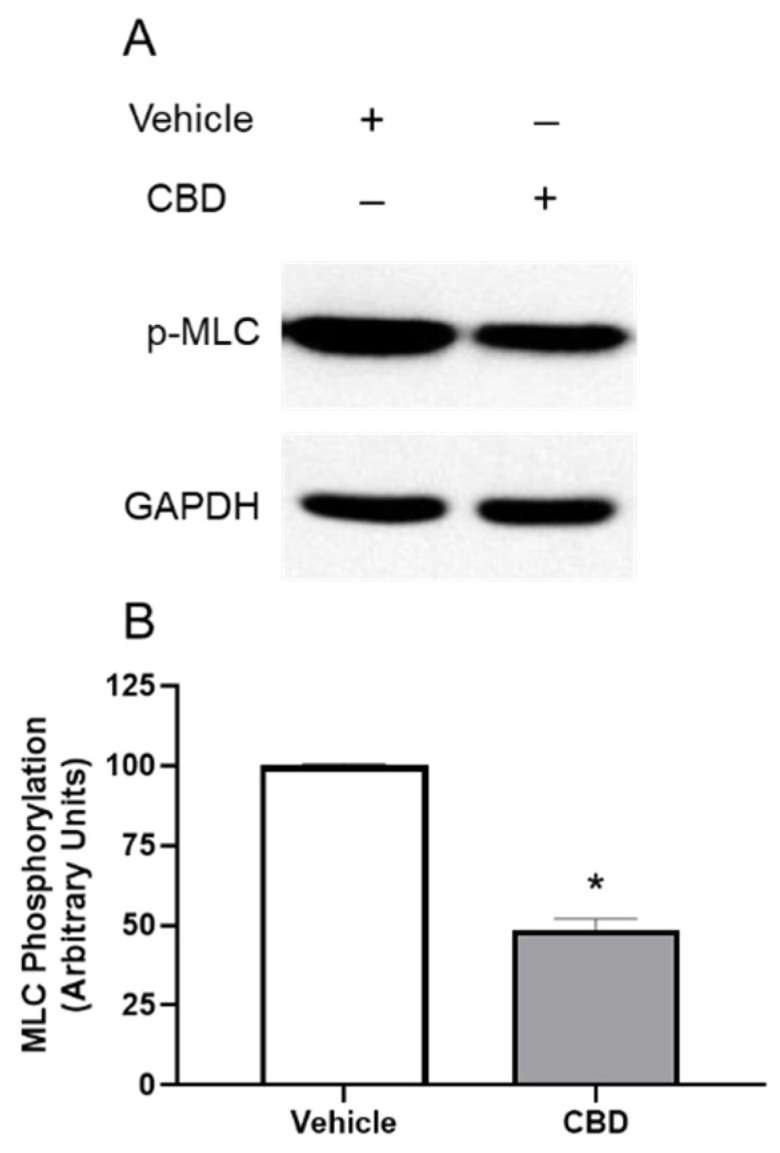
Inhibition of phosphorylation of myosin light chain protein by cannabidiol in trabecular meshwork cells. (**A**): Western blot representative of results obtained in three experiments. Cells were serum starved overnight and treated with vehicle or 1 µM CBD for 2 h, then phosphorylation of myosin light chain (MLC) was measured with anti-GAPDH and anti-phospho-MLC antibodies. (**B**): densitometry quantification of Western blot data from three experiments. MLC is constitutively phosphorylated in vehicle treated TM; following treatment with CBD, MLC phosphorylation significantly decreased. Results are expressed as mean ± SEM (*n* = 3). Vehicle treated phosphorylation of MLC levels are normalized to 100%. * Significant difference from vehicle determined by *t*-test, *p* < 0.05.

To determine the constitutive level of MLC phosphorylation and whether CBD stimulation would alter phosphorylation of MLC in TM cells, cells were serum starved overnight followed by treatment with vehicle, or CBD (1 μM) for 2 h. As shown in Figure 3, MLC is constitutively phosphorylated in TM cells. Following treatment with CBD, MLC phosphorylation significantly decreased by 51.56% (mean ± SEM: 48.44 ± 6.268; *p* < 0.05). These results demonstrate that in TM cells CBD inhibits MLC phosphorylation.

### 3.4. The Effects of CBD on MYPT1 Phosphorylation in TM Cells

**Figure 4:**
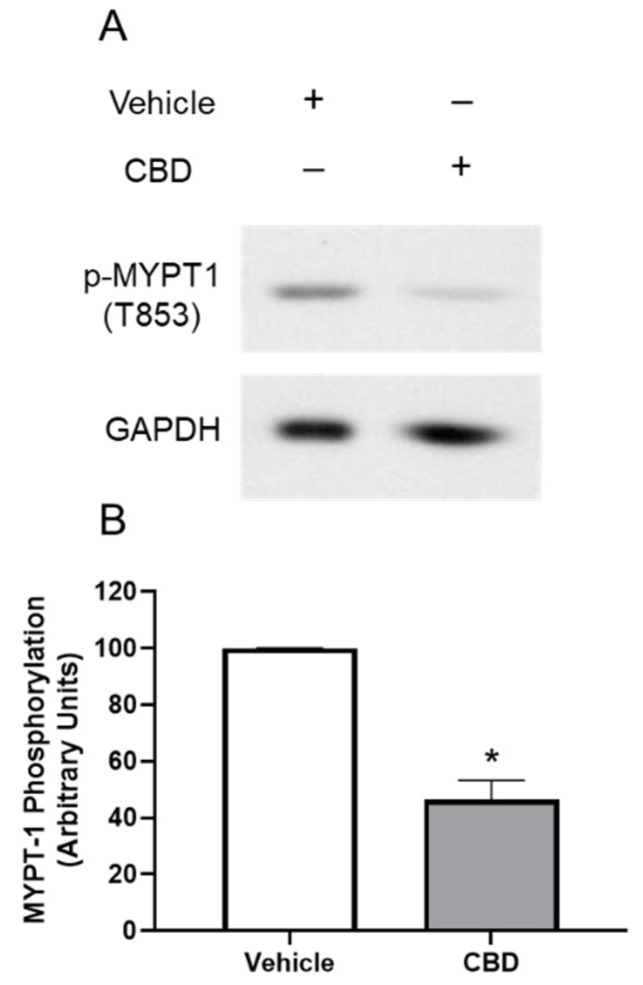
Effect of cannabidiol on myosin phosphatase targeting subunit 1 (MYPT1) phosphorylation. (**A**): Western blot representative of phosphorylation of myosin phosphatase targeting subunit 1 (MYPT1) at Thr853 for three experiments is shown. TM cells were serum starved overnight and treated with vehicle or 1 µM CBD for 2 h, then phosphorylation of MYPT1 at Thr853 was assessed. (**B**): densitometry quantification of phospho-MYPT1 from three experiments is shown. Following treatment with CBD, MYPT1 phosphorylation at Thr853 significantly decreased. Results are expressed as mean ± SEM (*n* = 3). Vehicle treated phosphorylation of MYPT1 levels are normalized to 100%. * Significant difference from vehicle determined by *t*-test, *p* < 0.05.

To determine if MYPT1 phosphorylation is altered by CBD, TM cells were serum starved overnight followed by treatment with DMEM medium or DMEM containing CBD (1 μM) for 2 h. As shown in Figure 4, following treatment with CBD, MYPT1 phosphorylation at Thr853 significantly decreased by 53.66% (mean ± SEM: 46.34 ± 11.95; *p* < 0.05). These results demonstrate that in TM cells CBD inhibits MYPT1 phosphorylation

### 3.5. The Effects of CBD on RhoA Activation in TM Cells

**Figure 5:**
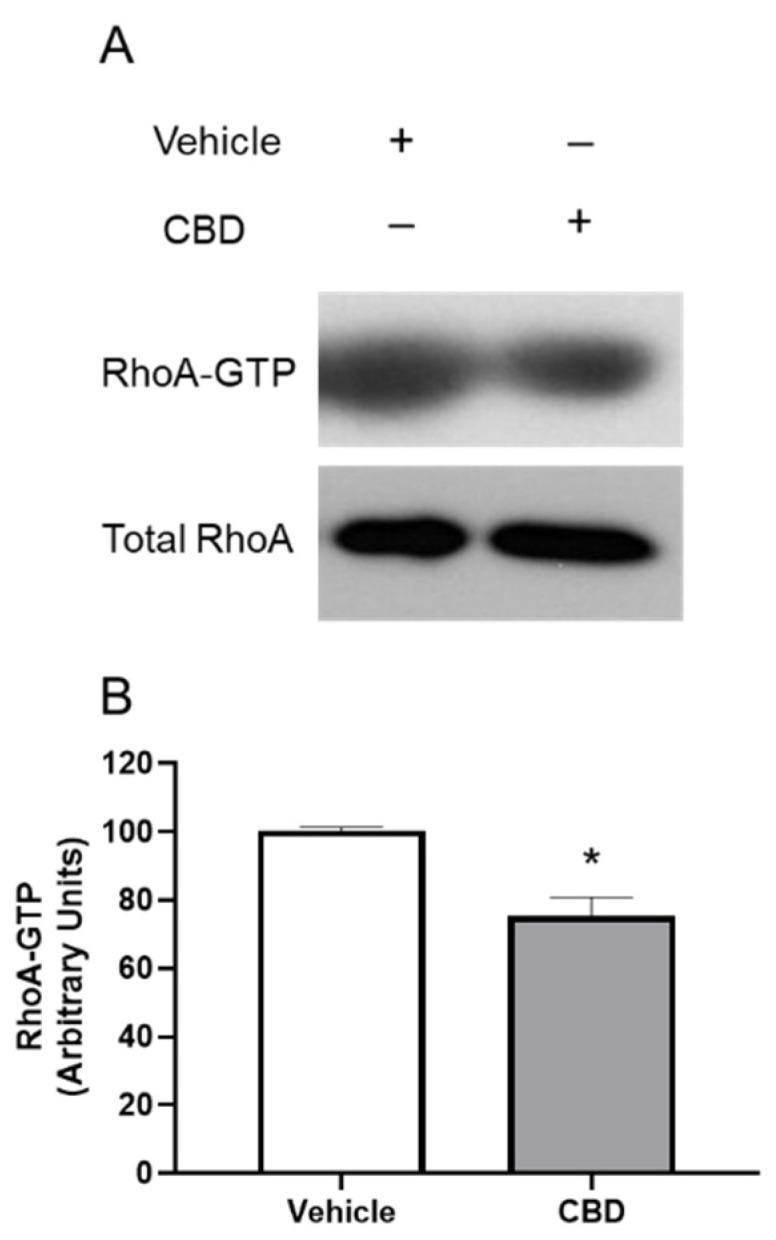
The effect of cannabidiol on RhoA activation. (**A**): representative Western blot of RhoA-GTP and Total RhoA for three experiments is shown. TM cells were serum starved overnight and treated with DMEM or DMEM containing 1 µM CBD for 2 h, then RhoA activation was assessed. (**B**): densitometry quantification of RhoA activation for three experiments is shown. RhoA-GTP significantly decreased following treatment with CBD compared to the vehicle in TM cells. Results are expressed as mean ± SEM (*n* = 3). Vehicle treated TM cell RhoA levels are normalized to 100%. * Significant difference from vehicle determined by *t*-test, *p* < 0.05.

To determine if RhoA activation is altered by CBD, the RhoA activation assay was performed. Cells were serum starved overnight followed by treatment with DMEM or DMEM containing CBD (1 μM) for 2 h. As shown in Figure 5, following treatment with 1 µM CBD, RhoA-GTP significantly decreased by 24.61% (mean ± SEM: 75.39 ± 9.539; *p* < 0.05). These results demonstrate that CBD inhibits RhoA activation in TM cells.

## 4. Discussion

IOP is maintained by balancing the production and outflow of aqueous humor [1,2,3]. In this study, using perfused porcine anterior segments, we demonstrated a CBD-induced significant increase in aqueous humor outflow. Therefore, our data support the notion that CBD lowers IOP through enhancing aqueous humor outflow. These results are consistent with previous preclinical studies that CBD was hypotensive when applied topically to cat or rabbit eyes [40,41]. Additionally, our data are consistent with a clinical report that CBD administered intravenously reduced IOP in human subjects [42].

In patients with primary open angle glaucoma, IOP elevation is caused by TM resistance to aqueous humor outflow associated with an excessive extracellular matrix accumulation, and an alteration of the TM cell contractility [3,4]. Previously, it has been shown that Rho/Rho kinase inhibitors enhance aqueous humor outflow through the TM route by decreasing the contractility of the TM cells [43]. In the current study, CBD was found to inhibit TM cell-mediated contraction of collagen gels. These data demonstrate that CBD was able to decrease the contractility of TM cells, supporting the role of CBD in enhancing aqueous humor outflow.

One of the signaling pathways for changing TM cell contractility is through regulation of myosin light chain (MLC) activity [21]. Previous studies have shown that aqueous humor outflow through the TM can be increased by inhibiting MLC phosphorylation [21]. In this study, administration of CBD led to an inhibition of MLC phosphorylation in TM cells. These data support the idea that CBD inhibit TM cell MLC activity, thus causing a decrease in TM cell contractility.

Dephosphorylation of MLC is induced by myosin light chain phosphatase (MLCP), which is heterotrimeric enzyme, containing a phosphatase subunit, a subunit with undefined function, and a regulatory subunit (MYPT1) [26,44]. Rho kinase itself is capable of phosphorylating MYPT1, thereby inactivating MLCP and inhibiting its phosphatase activity, and allowing sustained contraction [26,44]. In the current study, our results showed a decrease in MYPT1 Thr853 phosphorylation in response to CBD, indicating MLCP activation and MLC dephosphorylation.

Furthermore, our data demonstrated that RhoA activation was inhibited by CBD. This finding further supports the notion that CBD inhibits Rho/Rho kinase pathway, enhances dephosphorylation of MLC, and to the same end decreases the contractility of TM cells and allows for enhanced aqueous humor outflow.

In addition to having been approved for treating epilepsy in children, CBD is currently in clinical trials for a variety of diseases, including general pain and pain associated disorders, drug abuse and use disorders, other neurologic conditions and psychiatric conditions, and COVID-19 (from clinicaltrials.gov, accessed on 15 August 2022). Furthermore, there is an increasing interest by the public in the dietary supplement and potential therapeutic uses of CBD [45]. However, CBD use is not risk-free. Human studies have reported adverse effects of CBD including gastrointestinal issues, drowsiness, fatigue, and the most serious adverse side effect of CBD is elevated liver enzymes in liver function test [46,47].

Regarding the potential adverse effects for the eye, a recently published study in mice showed an increase in intraocular pressure (IOP) following topical application of a high concentration of CBD [48]. On the contrary, in another study also conducted in mice, CBD was shown to have no effects or decrease IOP, depending on the doses applied topically [49]. It is likely that the effects of CBD on IOP are dose related. Therefore, in the future it is important to examine the concentration-response of the CBD on aqueous humor outflow. In addition, in the future it is important to compare the efficacy of CBD with other drugs known to enhance aqueous humor outflow. Currently, the most frequently used route of administration of CBD for patients is oral intake of CBD oil. In the future it is crucial to study dose–response of CBD on IOP in patients to see if oral intake of CBD oil is beneficial or detrimental on IOP.

## 5. Conclusions

In summary, in this study we discovered, for the first time, that at 1 μM concentration CBD increases aqueous humor outflow in perfused anterior segments. In addition, using cultured TM cells, we demonstrated that CBD at 1 μM concentration inhibits TM cell contractility, MLC phosphorylation, MYPT1 phosphorylation and RhoA activation. Overall, our data support the concept that by altering the Rho/Rho kinase signaling to MLC, CBD was able to decrease the contractility of TM cells and enhance aqueous humor outflow via the TM route. There are many IOP-lowering drugs available to reduce aqueous humor production, but there are only limited drugs available to increase aqueous humor outflow directly through the TM route. This study demonstrated that as a potential therapeutic agent for lowering IOP, CBD is able to modify TM cell signaling and enhance aqueous humor outflow, which is often blocked in glaucoma.

## Data Availability

Not applicable.
